# Selective Oxidation of Cyclohexanone to Adipic Acid Using Molecular Oxygen in the Presence of Alkyl Nitrites and Transition Metals as Catalysts

**DOI:** 10.3390/ma16165722

**Published:** 2023-08-21

**Authors:** Dawid Lisicki, Beata Orlińska, Tomasz Martyniuk, Krzysztof Dziuba, Jakub Bińczak

**Affiliations:** 1 Department of Chemical Organic Technology and Petrochemistry, Silesian University of Technology, Akademicka 2A, 44-100 Gliwice, Poland; 2 Grupa Azoty Zakłady Azotowe “Puławy” S.A., Al. Tysiąclecia Państwa Polskiego 13, 24-110 Puławy, Poland; tomasz.martyniuk@grupaazoty.com (T.M.); krzysztof.dziuba@grupaazoty.com (K.D.); jakub.binczak@grupaazoty.com (J.B.)

**Keywords:** green chemistry, industrial synthesis, adipic acid, oxidation

## Abstract

This paper presents a not previously reported catalytic system consisting of transition metals Co^2+^ and Mn^2+^ and alkyl nitrites R-ONO for the oxidation of cyclohexanone with oxygen to adipic acid. The influence of type and amount of catalyst, temperature, time, and type of raw material on conversion and product composition were determined. In addition, the oxidation of selected cyclic ketones such as cyclopentanone, cyclohexanone, cyclooctanone, cyclododecanone, 2-methylcyclohexanone, 3-methylcyclohexanone, and 4-methylcyclohexanone in acetic acid as solvent was performed. The results showed that R-ONO systems, under established reaction conditions, form NO·radicals, which oxidize to NO_2_ under a strong oxidization reaction environment. The Co^2+^/Mn^2+^/NO_2_ system was shown to be highly active in the oxidation of cyclic ketones with oxygen.

## 1. Introduction

Aliphatic dicarboxylic acids are used, inter alia, in the production of polyamides, plasticizers, and polyesters. Dicarboxylic acids such as adipic acid (AA), glutaric acid (GA), cork, and 1,12-dodecanedioic acid are mainly produced via the oxidation of cyclic ketones and/or alcohols with HNO_3_ [1]. 1,10-decanedioic acid is obtained by esterification of AA, followed by electrolysis and hydrolysis [2]. The methods of dicarboxylic acids production through oxidative cleavage of unsaturated carboxylic acids or hydrogenation of unsaturated diacids are also practiced on industrial scale [3].

**Figure 1:**
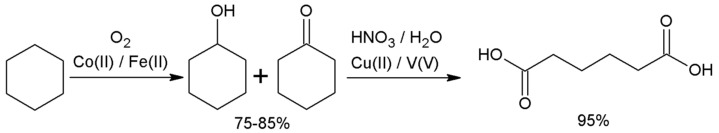
Industry method of AA production from cyclohexane.

Undoubtedly, in industry, AA is considered the most important dicarboxylic acid. In 2014, the AA market was valued at USD 6.5 billion [4], and world production was 2.7 million tons. It has been industrially manufactured since the 1940s via Du Pont technology from cyclohexane [5]. In the first stage of this method, cyclohexane is oxidized to a mixture of cyclohexanol (C-OL) and cyclohexanone (C-ON) with air and in the presence of 0.01–1 ppm Co^2+^/Fe^2+^ (conversion 4–8%, selectivity 70–80%, 0.5–2.0 MPa, 140–180 °C) [6]. In the next stage, the obtained mixture is converted to AA via oxidation with HNO_3_ (Figure 1) [7,8].

One of the by-products of this process is N_2_O, which is difficult to manage and utilize because it is a powerful greenhouse gas, approx. 300 times stronger than CO_2_, with a half-life of approx. 120 years [9]. As a result of AA production, about 290–310 kg of N_2_O/t_AA_ is obtained [10].

Currently, it is desirable to develop an ecological and economically viable method for the production of dicarboxylic acids [11] that meets specific requirements of sustainable development and principles of green chemistry (high conversion, selectivity, heterogeneous catalysis, use of renewable raw materials [12,13,14,15,16], and elimination of solvents). However, designing technologies that adhere to such restrictions is extremely difficult. As shown previously [17], AA production from biomass is associated with higher greenhouse gas emissions and higher energy consumption compared to traditional processes using benzene as a petrochemical raw material. Therefore, the replacement of the non-ecological oxidizing agent HNO_3_ is the most important issue in dicarboxylic acid production.

The literature has described oxidation processes of cyclohexane directly to AA, with oxygen/air, O_3_ [18]_,_ and HNO_3_ [19], with the main focus being the use of oxygen/air as the oxidizing agent. The reaction is mainly carried out in acetic acid (AcOH) as a solvent and in the presence of transition metal salts or complexes [20]. In order to initiate the process, small amounts of cyclohexanone (C-ON) are introduced [21,22]. Additionally, reports have utilized oxidative organocatalysts such as *N*-hydroxyphthalimide (NHPI) [23,24,25,26,27,28,29] and but-2-one (MEK) [30]. Importantly, the oxidation of cyclohexane to AA in the absence of solvent has been studied in the presence of heterogeneous catalysts such as Fe(III)AlPO-31 [31], NHPI/Fe(BTC) [32], Fe(III)T(O-Cl)PP [33]^,^ and salts as well as complexes of transition metals in combination with a lipophilic 4-dodecyloxycarbonyl-*N*-hydroxyphthalimide (C_12_-NHPI) derivative [34]. Oxidation of cyclohexane has been also achieved using a AcOH/H_2_O mixture in the presence of Co/Mn [35] and Mn(III)T(p-Cl)PP [36] clusters in PhCOOH/H_2_O [36] and AcOH/scCO_2_ [37].

It is of particular interest to replace HNO_3_ in the oxidation of C-ON and/or C-OL to AA with environmentally friendly oxidizing agents such as oxygen (O_2_), air, or H_2_O_2_, which eliminates problems related to the management or utilization of the generated NO_x_ [38]. The use of environmentally friendly oxidizing agents requires the development of catalytic systems capable of obtaining AA with high selectivity. The first patents related to C-ON oxidation under air to AA in the presence of transition metal salts appeared in the 1930s and 1940s [39,40,41,42,43]. Table 1 shows examples of catalysts used in the oxidation of C-ON to AA with O_2_ or air.

The process of oxidation of C-ON to AA in the presence of O_2_ or air was examined previously [53]. The oxidation reaction was carried using transition metals, mainly Co^2+^ and Mn^3+^ acetates or acetylacetonates and para-toluenesulfonic acid (p-TS). The researchers found that the presence of p-TS improved the selectivity of the reaction. For example, when C-ON oxidation was conducted in AcOH (1:7.3 *v*:*v*) in the presence of H_2_O and using the Co^2+^/Mn^2+^ system with p-TS, AA was obtained with 78% selectivity and 99.3% C-ON conversion. The reaction conditions were 5 h reaction time, 1.21 MPa pressure, at 70 °C using a mixture of O_2_ (4.9 L/h) and N_2_ (48.1 L/h). Additionally, high AA yields have been obtained via oxidation of C-ON with O_2_ in the presence of Mn(NO_3_)_2_/Co(NO_3_)_2_ [54].

Recently, the beneficial effect of nitrites on the oxidation of hydrocarbons with O_2_ has been reported [56]. The authors showed that isoamyl nitrite (IPN) increased the conversion of cycloalkanes (C_5_–C_8_) via oxidation with O_2_ in the presence of the system Co(acac)_2_ and or Mn(acac)_2_. Others have shown the oxidation of olefins with O_2_ using tert-butyl nitrite (TBN) [57,58,59]. Additionally, reports have shown the oxidation of alcohols to aldehydes and/or ketones with O_2_ using TBN in systems with DDQ (2,3-dichloro-5,6-dicyano-1,4-benzoquinone) [60], TEMPO/HBr ((2,2,6,6-tetramethyl piperidin-1-yl)oxyl) [61], TEMPO/DMC (dichloromethane) [62], 4-OH-TEMPO/TCQ/HCl ((4-hydroxyl (2,2,6,6-tetramethyl piperidin-1-yl)oxyl)/tetrachlorobenzoquinone) [63], MNST/H_2_O (2,2,6,6-tetramethylpiperidin-1-yl)oxyl on Fe_3_O_4_ silicate nanoparticles) [64], IL@SBA-15-TEMPO/AcOH (SBA-15 functionalized, with TEMPO and ionic liquid) [65], DDQ/AcOH/DMC [66], ABNO/KPF_6_/H_2_O (8-azabicyclo [3.3.1] nonagn-*N*-oxyl) [67], NHPI/MeCN [68], and AZADO/MeCN (2-azaadamantane-*N*-oxyl) [69].

In this study, the influence of alkyl nitrites on the oxidation of cyclic ketones to dicarboxylic acids was demonstrated for the first time. The beneficial effect of the addition of nitrites such as pentyl- (IP), tert-butyl- (TBN), and isopentyl (IPN) nitrite in a system with Co^2+^ and Mn^2+^ on AA selectivity and C-ON conversion was described. The obtained results show that R-ONO systems form NO· radicals, which oxidize to NO_2_ in a strongly oxidizing environment. The proposed Co^2+^/Mn^2+^/NO_2_ system provided high activity in the oxidation of cyclic ketones with O_2_.

## 2. Materials and Methods

### 2.1. Materials

The following were used: cyclopentanone (Sigma-Aldrich, St. Louis, MI, USA, ≥99%), cyclohexanone (Sigma-Aldrich, ≥99%), cycloheptanone (Sigma-Aldrich, ≥99%), cyclooctanone (Sigma-Aldrich, ≥98%), cyclododecanone (Merck, Rahway, NJ USA, ≥98%), 2-methylcyclohexanone (Aldrich, St. Louis, MI, USA, ≥98%), 3-methylcyclohexanone (Aldrich, ≥97%), 4-methylcyclohexanone (Aldrich, ≥99%), cyclohexanol (Sigma-Aldrich, >99%), cyclohexane (Sigma-Aldrich, >99%), manganese(II) acetylacetonate (Sigma-Aldrich, 99%), cobalt(II) acetylacetonate (Sigma-Aldrich, 97%), manganese(II) acetate tetrahydrate (Sigma-Aldrich, 99.99%), cobalt(II) acetate tetrahydrate (Sigma-Aldrich, 99.99%), manganase(II) nitrate tetrahydrate (Sigma-Aldrich, ≥97%), cobalt(II) nitrate hexahydrate (Sigma-Aldrich, >99%), isopentyl nitrite (Sigma-Aldrich, 96%), tert-butyl nitrite (Sigma-Aldrich, 90%), N-hydroxyphthalimide (Acros, Waltham, MA, USA, 98%), methyl ethyl ketone (Sigma-Aldrich, 99.7%), nitric acid (Sigma-Aldrich, 60%), pentyl nitrite (Sigma-Aldrich, 98%), acetic acid (Chempur, Karlsruhe, Germany, 99.5%), acetonitrile (Chempur, 99.5%), benzonitrile (Sigma-Aldrich, 99%), sulfuric acid (Chempur, 98%), and methanol (Chempur, 99.8%).

### 2.2. General Procedure for Catalytic Oxidation under Pressure in 100 mL Volume

The 100 mL oxidation process was carried out in an Autoclave Engineers Inc. pressure reactor, (Erie, PA, USA), made of Hastelloy C-276 steel and equipped with a high-speed stirrer, heating jacket, internal cooler, and reflux condenser. In a typical process, 2 mL of substrate, 20 mL of solvent, and the catalytic system were introduced into the reactor. The reactor contents were purged with O_2_ at 2 L/h for 2 min, then heated to 40–100 °C and stirred at 1000 rpm. Subsequently, O_2_ was introduced into the reactor under pressure 0.1–1.5 MPa, and the oxidation process was started. The pressure, the stirring speed and temperature were monitored using a Sentinel control device. During the process, the pressure decreased due to O_2_ consumption and was supplemented with additional O_2_.

### 2.3. General Procedure for Catalytic Oxidation under Pressure in 600 mL Volume

The oxidation process on 600 mL scale was carried out in a PARR pressure reactor (Moline, IL, USA) made, of Hastelloy C-276 steel, equipped with a high-speed stirrer, heating oil jacket, internal cooler, and reflux condenser. Air was introduced into the reactor through a bubble placed under the agitator. In a typical process, 30 mL of C-ON, 300 mL of AcOH, and a catalytic system were introduced into the reactor. The reactor contents were purged with air or O_2_ at 50 L/h for 2 min, then heated to 60 °C and stirred at 600 rpm. Subsequently, the oxidizing agent was introduced into the reactor at a pressure of 0.5 MPa, the stirring speed was increased to 1200 rpm, the oxidizing agent blow-through was set to 50 L/h, and the oxidation process was started. The pressure, stirring speed and temperature were monitored using a PARR control device. During the oxidation process, the amount of oxidizing agent and gases introduced and oxygen concentration in the gases were monitored. This information was used to calculate the amount of consumed oxygen.

### 2.4. General Procedure for Catalytic Oxidation under Atmospheric Pressure

The oxidation process on 120 mL scale was carried out in a thermostatic bubble reactor made of glass and equipped with a G5 glass bubble and reflux condenser. Air was introduced into the reactor through a bubble placed at the bottom of the reactor.

In a typical process, 8 mL of C-ON, 80 mL of AcOH, and a catalytic system were introduced into the reactor. The reactor contents were heated to 60 °C. Subsequently, the oxidizing agent (air) was added into the reactor at a flow of 12 L/h. During the oxidation process, the amount of oxidizing agent added introduced to the reactor and the amount of oxygen in the off-gas were monitored.

### 2.5. Analytical Methods

The reaction products were analyzed using an Agillent 5890 Series II gas chromatograph equipped with an FID detector, Zebron ZB-5HT column (30 m × 0.25 mm × 0.25 µm) and automatic sample dispenser. Helium was used as the carrier gas. The analysis was performed using the standard method with an internal standard (toluene) (injection port temperature 200 °C, detector temperature 300 °C, split 100:1, injection 1 μL, air 400 mL/min, nitrogen 24 mL/min, hydrogen 30 mL/min, temperature program 70 °C for 10 min, 6 °C/min to 112 °C, 20 °C/min to 212 °C, 8 min at 212 °C). Each sample was analyzed twice, and the substance concentration was calculated on the basis of previously prepared standard curves. The products composition was additionally confirmed by gas chromatography coupled to mass spectrometry (GC-MS) performed using an Agilent gas chromatograph 7890C (Agilent HP-5 MS capillary column, 30 m × 0.25 mm × 0.25 μm, helium 1 mL/min) coupled with an Agilent mass spectrometer 5975C with EI ionization (70 eV) using the NIST/EPA/NIH Mass Spectral Library.

Raw material conversion: First, 1 mL of sample was taken from the reaction products, and 5 mL of toluene in AcOH solution (6 g of toluene in 250 mL of acetic acid) was added. The prepared solution was analyzed by GC.

Content of dicarboxylic acids: Next, 1 mL of sample was taken from the reaction products, and 12 mL of a toluene/methanol solution (12 g of toluene in 1000 mL of methanol) and 1–3 drops of sulfuric acid (VI) were added. The solution was stirred (200 rpm) for 24 h at ambient temperature to form the methanol esters from carboxylic acids. The prepared solution was analyzed by GC, which determined the amount of dimethyl esters of dicarboxylic acids.

It was found that the error resulting from reproducibility and repeatability amounted to 2%.

## 3. Results

### 3.1. Oxidation of C-ON with O_2_ to AA Using the Co^2+^/Mn^2+^/R-ONO System

**Figure 2:**
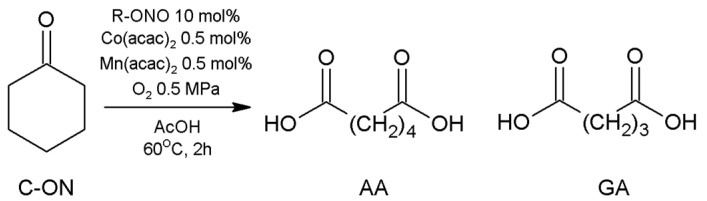
Aerobic oxidation of C-ON.

Research was carried out on the oxidation reactions of C-ON to AA using alkyl nitrites R-ONO in the Co^2+^/Mn^2+^ system and, in polar solvents (AcOH), acetonitrile (MeCN) or benzonitrile (PhCN)). IPN, amyl nitrite (PN), and TBN were used in the research. A reaction was performed using HNO_3_ for comparison. In the reaction products, the content of C-ON (AA) and the main by-product (GA) was determined. The reaction was carried out at 50–120 °C, for 2–6 h, under pressure of 0.5–1.5 MPa O_2_ (Figure 2). The results of the various experiments are presented in Table 2.

The results show that under the tested conditions (60 °C, 0.5 MPa O_2_), the addition of each R-ONO increased C-ON conversion. Addition of IPN and TBN increased AA selectivity (entries 5, 7), where the IPN/Co^2+^/Mn^2+^ system gave 62% selectivity and 97% C-ON conversion (entry 5). In the case of the Co^2+^/Mn^2+^ system, AA was obtained with 42% selectivity and 68% C-ON conversion (entry 1).

Interestingly, the C-ON oxidation process occurred even under mild reaction conditions (60 °C) and catalyzed solely with IPN (entry 2). This highlights IPN high activity as an organocatalyst. To our knowledge, this is the first example of oxidation of cyclic ketones without the participation of metal catalysts. Examination of Co^2+^ and Mn^2+^ compounds revealed no significant differences in reaction outcome (entries 9, 10). Furthermore, addition of HNO_3_ increased C-ON conversion and AA selectivity (entry 8). However, the IPN/Co^2+^/Mn^2+^ system provided AA in higher selectivity. The observed variation in selectivity may stem from NO_2_ generation from R-ONO and HNO_3_, where R-ONO at 60 °C decomposes to R-O⸱ and NO·. However, under strongly oxidizing conditions, NO· undergoes spontaneous oxidation to NO_2_, which contributes to the oxidation reaction. In the case of HNO_3_, an equilibrium forms between NO_2_, O_2_, and H_2_O, and under high pressure (0.5 MPa), this equilibrium shifts towards HNO_3_, which results in lower activity of the HNO_3_/Co^2+^/Mn^2+^ system.

**Figure 3:**
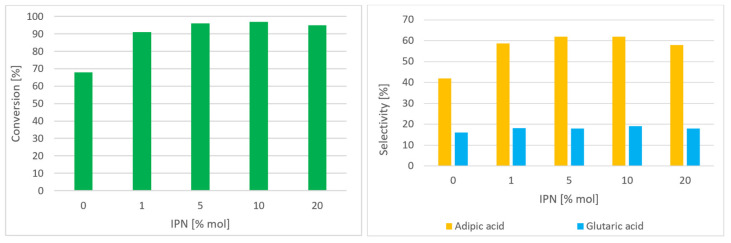
Influence of the amount of IPN on the composition of reaction products C-ON 2 mL (19.3 mmol), AcOH 20 mL (349.7 mmol), IPN 10% mol, Co(acac)_2_ 0.5% mol, Mn(acac)_2_ 0.5% mol at 60 °C, and oxygen 0.5 MPa at 2 h.

In the case of the IPN/Co(acac)_2_/Mn(acac)_2_ catalytic system, which generates AA in the highest selectivity, the influence of basic parameters on the composition of C-ON oxidation reaction products was determined. Figure 3 shows the influence of IPN amount (1–20 mol%) on C-ON conversion and AA and GA selectivity. For comparison, the reaction without IPN addition was conducted.

The addition of 1 mol% of IPN increased C-ON conversion from 68% to 91% and AA selectivity from 42% to 59%. When the amount of IPN increased to 5 mol%, AA was obtained in 62% selectivity and 96% conversion. Any further increase in IPN amount (20 mol%) had no significant affect the composition of the reaction products.

Table 3 lists the experimental results examining the influence of temperature, O_2_ pressure and the type of solvent on the composition of the products of C-ON oxidation to AA in the IPN/Co^2+^/Mn^2+^ system (Table 3).

The obtained results showed that as the temperature increased from 40 to 80 °C (entries 1–4), C-ON conversion increased from 52% to 100% and AA selectivity from 31% to 68%. However, when the temperature increased to 100 °C, AA selectivity deceased (entry 5). At higher temperature, the share of the oxidation reaction to by-products such as lower carboxylic acids was greater, as evidenced by the greater selectivity of the GA obtained. The oxidation was carried out at 60 °C for the first 1 h and at 80 °C for the second h (entry 6). This resulted in similar C-ON conversion and AA selectivity to the reaction conducted at 80 °C (entry 4). Increasing O_2_ pressure above 0.1 MPa increased the rate of C-ON oxidation reaction; however, as expected, it did not significantly affect AA selectivity (entries 3, 7–9). According to the results, the role of O_2_ was more relevant when considering air or O_2_-enriched air oxidation. Elimination of solvent from C-ON oxidation or oxidation conducted in acetonitrile decreased C-ON conversion and significantly decreased AA selectivity (entries 10–12). C-ON oxidation conducted in PhCN gave a conversion of 99% and 64% AA selectivity (entry 13). This approach can be advantageous for industry due to the reduced effect of the reaction medium on the corrosive effect compared to oxidation in AcOH. Hence, the use of PhCN in an industrial process is more desirable due to less corrosion of equipment.

**Figure 4:**
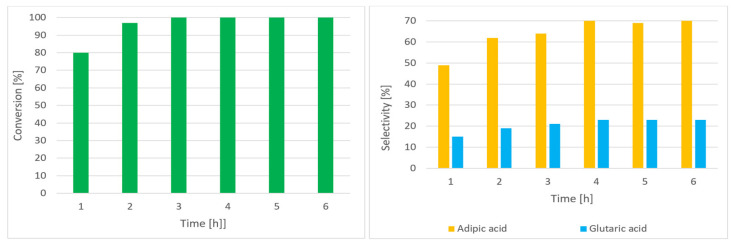
The effect of time on the composition of reaction products C-ON 2 mL (19.3 mmol), AcOH 20 mL (349.7 mmol), IPN 10 mol%, Co(acac)_2_ 0.5 mol%, Mn(acac)_2_ 0.5 mol% at 60 °C, and O_2_ 0.5 MPa.

Figure 4 shows the effect of time on C-ON conversion and AA and GA selectivity (1–6 h).

Under the reaction conditions stated in Figure 4, C-ON conversion was 100% after 3 h. It was found that increasing the reaction time from 1 to 4 h gradually increased AA selectivity from 49% to 71%. Further extension of the reaction time did not affect the composition of the products.

### 3.2. Oxidation of Cyclohexanol, C-ON, or Their Mixtures with O_2_ Using the IPN/Co(acac)_2_/Mn(acac)_2_ System

As a result of the industrial process of cyclohexane oxidation, a mixture of C-ON and C-OL was formed. Therefore, the possibility of using C-OL or a mixture of C-OL and C-ON in the AA preparation process was investigated in relation to the tested IPN/Co(acac)_2_/Mn(acac)_2_ system (Table 4).

On the basis of the above results, the main product of the C-OL oxidation reaction was C-ON, and AA was obtained in relatively low selectivity of 11% and 20% after 2 h and 6 h, respectively (entries 3, 4). Using 1:1 (*w*:*w*) mixture of C-ON and C-OL as the substrate, AA was obtained with 58% selectivity, with 100% conversion of raw materials (entry 5). Prolongation of the oxidation reaction to 6 h only gave a slight increase in AA selectivity (entry 6). It has been reported that the oxidation rate of cyclic alcohol is much lower than that of cyclic ketone, mainly due to the large amounts of C-ON in the mixture used to initiate the oxidation of C-OL to AA.

### 3.3. Study of the Oxidation of Cyclic Ketones Using the IPN/Co(acac)_2_/Mn(acac)_2_ System

The possibility of using the proposed catalytic system in the oxidation of a series of cyclic ketones (C_5_–C_12_) in order to form the appropriate dicarboxylic acids (C_5_–C_12_) was examined. The reactions were carried out at 60 °C and 80 °C in the presence and absence of 10 mol% IPN (Table 5).

According to the results listed in Table 5, when the temperature was at 80 °C with the addition of 10 mol% IPN, an increase in selectivity was observed for all appropriate dicarboxylic acids. Additionally, the addition of 10 mol% IPN to the oxidation of cycloheptanone, cyclooctanone, cyclododecanone, 2-methylcyclohexanone, 3-methyl cyclohexanone, and 4-methylcyclohexanone increased conversion.

The catalytic activity of the IPN/Co(acac)_2_/Mn(acac)_2_ system was also productive at 60 °C. Lowering the reaction temperature from 80 °C to 60 °C did not affect the conversion of C-ON (entry 6), cyclododecanone (entry 15), 2-methylcyclohexanone (entry 18), and 3-methylcyclohexanone (entry 21); however, a slight reduction was observed for cyclopentanone (entry 3), cyclooctanone (entry 12), and 4-methylcyclohexanone (entry 24). Furthermore, a noticeable reduction in conversion from 99% to 47% was detected for cycloheptanone. Additionally, reducing the temperature adversely affected the selectivity (entries 3, 9, 12, 15, 24) except for AA (entry 6), 6-oxyheptanoic acid (entry 18), and 3-methyladipic acid (entry 21). The main by-products observed in the reaction were shorter-chain dicarboxylic acids.

### 3.4. C-ON Oxidation with Air Using the IPN/Co(acac)_2_/Mn(acac)_2_ System

**Figure 5:**
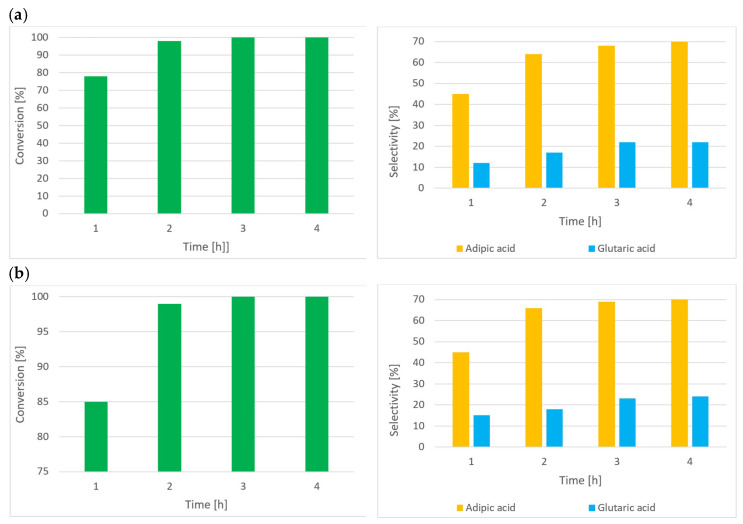
Air or O_2_ oxidation of C-ON. C-ON 30 mL (0.29 mol), AcOH 300 mL (5.25 mol), IPN 10 mol%, Co(acac)_2_ 0.5 mol%, Mn(acac)_2_ 0.5 mol% at 60 °C, and 0.5 MPa. (**a**) Air 50 L/h; (**b**) oxygen 50 L/h.

Industrial oxidation processes using O_2_ as an oxidizing agent are associated with a greater risk of explosion due to the formation of a wide range of explosive mixtures between the raw material, solvent, and O_2_ compared to air. The oxidation reaction was carried out at a flow of 50 L/h using the IPN/Co^2+^/Mn^2+^ system (Figure 5). Therefore, the oxidation process of C-ON with air and was compared to oxidation using O_2_.

The results showed that under the tested reaction conditions, the type of the oxidizing agent used (air or O_2_) had no significant influence on the composition of C-ON oxidation reaction products. However, when the reaction was conducted in O_2_, the rate of C-ON conversion and AA and GA formation was slightly higher after the first 2 h of the reaction. Differences in the reaction rate depended on the oxidizing agent used (air or O_2_) and may also have depended on the design of the reactor and distribution system of the oxidizing agent in the liquid reaction mixture.

**Figure 6:**
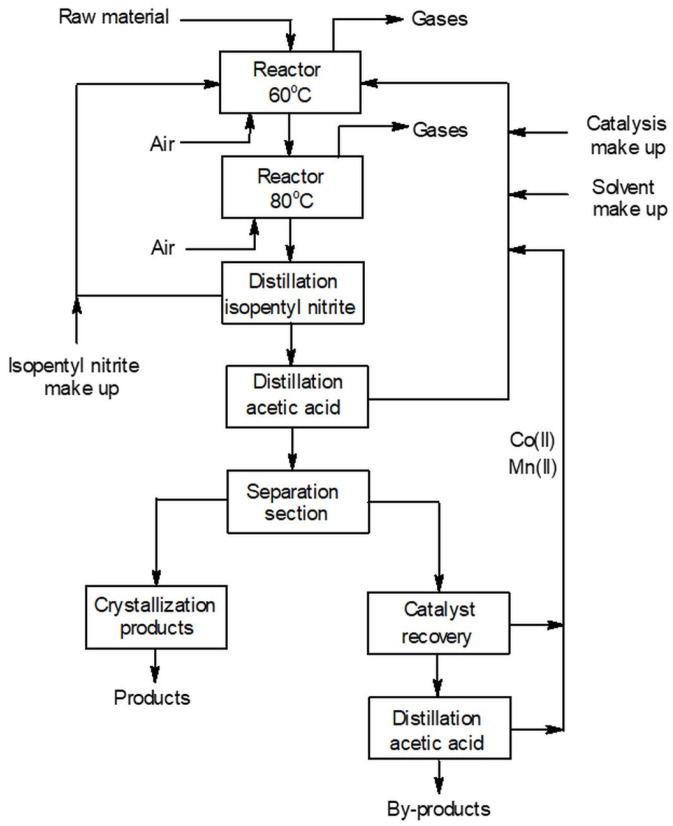
Flowchart for the preparation of carboxylic acids from cyclic ketones.

Figure 6 shows the proposed block diagram of the process for the production of appropriate dicarboxylic acids from cyclic ketones based on our optimized conditions. The figure includes sections for reaction, product isolation and purification, and solvent and catalyst recycling.

It is known that the oxidation of cyclic ketones in the presence of O_2_ is very exothermic. In the case of 330 mL scale in a 600 mL reactor, it was necessary to cool the reaction mixture in order to maintain a constant temperature. Therefore, a solution to this issue may be the use of a reaction system consisting of a cascade of two reactors. As a result, it was possible to carry out the oxidation at a lower rate in 1onereactor (60 °C), which facilitated the controlled heat removal. The calculated heats of oxidation reactions of C-ON to AA, GA, and SA are 748.9, 1367.3, and 2026.9 kJ/mol, respectively. Taking into account the selectivity of respective reactions (AA = 70%, GA = 22%, SA = 8%) and a 100% conversion of C-ON, the total heat of the oxidation process can be estimated at 987.2 kJ/mol. Our research has shown that the purpose of the second reactor was to increase the selectivity of the reaction via oxygenation of the intermediates. It was therefore advantageous to use a higher temperature of about 80 °C.

The next steps were to separate IPN and part of the solvent (AcOH) from the reaction products. According to the results obtained for the oxidation of C-ON to AA, the concentration of the reaction products by evaporation to approx. 80% AcOH enables separation of AA with 80% efficiency. Upon reduction of the temperature, a precipitate was formed mainly composed of AA and impurities such as glutaric and succinic acid. AA was further purification by crystallization from AcOH, water, or a mixture thereof. Depending on the type of dicarboxylic acid employed, it was necessary to evaporate different volumes of AcOH due to differences in solubility. The last steps involved the separation and recycling of the Co^2+^ and Mn^2+^ catalyst and the regeneration of residual AcOH.

### 3.5. Proposed Mechanism for the Oxidation of C-ON with O_2_ Using the IPN/Co(acac)_2_/Mn(acac)_2_ System

**Figure 7:**
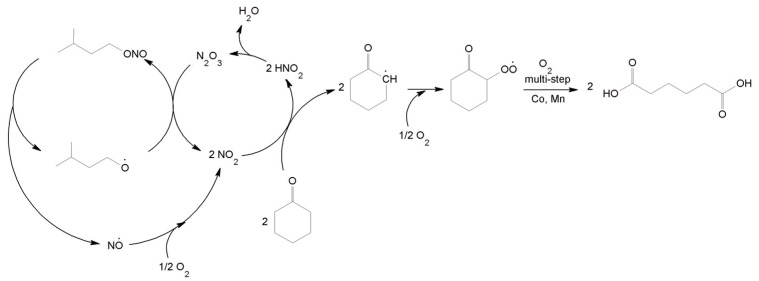
Probable mechanism for the oxidation of C-ON to AA.

The current literature has suggested that the addition of alkyl nitrites to the oxidation reaction of cyclic ketones increases the reaction rate, which allows for higher conversions and selectivity at lower temperatures. Figure 7 shows the proposed mechanism of the oxidation of cyclic ketones using the IPN/Co^2+^/Mn^2+^ system.

We propose that during the reaction, due to temperature, IPN decomposed to isopentyl radical and NO·. NO· radical was then oxidized to NO_2_ under O_2_, which initiated the oxidation of C-ON to form 2-carbonylcyclohexyl radical and HNO_2_. Then, as a result of oxidation, reduction, and disproportionation, the isopentyl radical transformed into IPN [70]. The exact course of AA formation from 2-carbonylcyclohexyl radical was presented in our previous paper [21].

We confirmed the NO_2_ catalytic activity by carrying out C-ON oxidation in AcOH, 0.2 mol% Mn(acac)_2_, 0.2 mol% Co(acac)_2_, and 10 mol% NO_2_ (60 °C, air 12 L/h). NO_2_ gas produced in a separate reaction vessel by dropping HNO_3_ into Cu was redirected to the oxidation reactor along with air. After the brown NO_2_ was dissolved in the reaction mixture, a color change was observed from yellow to dark brown as well as O_2_ consumption, which was monitored by measuring the O_2_ concentration in the gases exiting the reactor. The results showed that the O_2_ concentration dropped from 21% to 10% in just 5 min. The reaction continued for a further 2 h.

**Figure 8:**
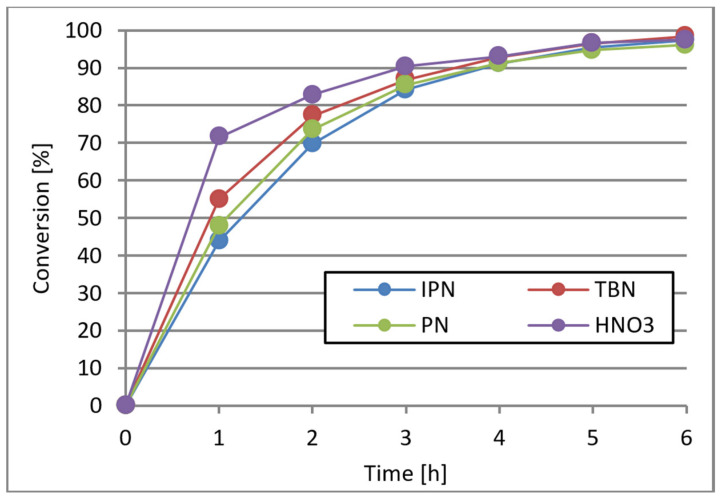
Influence of NO_2_ initiator on C-ON conversion at atmospheric pressure. C-ON 8 mL, AcOH 80 mL, Co(acac)₂ 0.2 mol%, Mn(acac)₂ 0.2 mol%, initiator 10 mol%, air 12 L/h, and 0.1 MPa at 60 °C.

The water present in the reaction system reacted with NO_2_ to form HNO_3_ and HNO_2_. The produced HNO_2_ molecule completed the catalytic cycle, and the presence of HNO_3_ (Table 2, entry 10) influenced the oxidation process. The catalytic activity of HNO_3_ in the reaction system was largely related to the pressure. As described earlier, HNO_3_ formed an equilibrium with NO_2_, O_2_, and H_2_O. Under increased pressure (0.5 MPa), the equilibrium shifted towards HNO_3_, which resulted in lower activity of the HNO_3_/Co^2+^/Mn^2+^ system. However, at atmospheric pressure, the activity of the HNO_3_/Co^2+^/Mn^2+^ system was significantly higher (Figure 8).

Each of the selected catalytic systems was highly active in the C-ON oxidation process. The rate of C-ON oxidation in the presence of HNO_3_ was initially faster in comparison to IPN, PN, and TBN. However, it was reduced after 1h. As a result, after 4 h, C-ON conversion was similar in all cases, ranging from 91–92%. After 6 h of reaction, 98% conversion was obtained for all tested catalytic systems.

**Figure 9:**
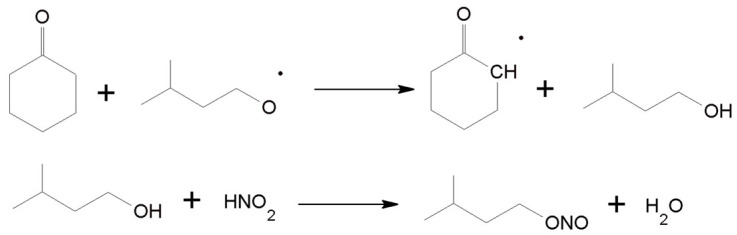
Removal of the hydrogen atom from C-ON via isopentyl radical.

GC/MS analysis was utilized to confirm the negligible amounts of isopentyl alcohol in the reaction products, which may prove the detachment of the hydrogen atom from the molecule, e.g., C-ON, by the isopentyl radical (Figure 9). A similar mechanism of the catalytic effect of IPN was proposed by researchers when examining the oxidation of cyclohexane directly to AA^55^. The isopentyl radical removes the hydrogen atom from cyclohexane, producing cyclohexyl radical and isopentyl alcohol, which, as a result of slow conversion, generates IPN via the reaction with HNO_2_. Interestingly, only small amounts of isopentyl alcohol were observed in our reaction products even when they were conducted using 20 mol% IPN. Therefore, only slight conversion of IPN to alcohol occurs under the proposed reaction conditions.

Additionally, it may be also possible to detach the hydrogen atom from C-ON via radical NO· to form 2-carbonylcyclohexyl radical and HNO. The presence of HNO in the reaction system could promote N_2_O formation as a by-product. Reports have shown that HNO can undergo dimerization to form N_2_O and H_2_O or react with the NO_2_ present in the system, which generates NO and HNO_2_. However, the possible reactions leading to N_2_O were insignificant due to the low probability of HNO formation in the highly oxidizing reaction medium. Moreover, HNO dimerization was unlikely due to its trace amounts in the reaction system.

## 4. Conclusions

Herein, we found a positive effect of the addition of IPN, TBN, PN, and HNO_3_ towards the composition of the products resulting from C-ON oxidation to AA in air. In the presence of the IPN/Co^2+^/Mn^2^+ catalytic system, AA was obtained in the highest selectivity. Additionally, the anion type in the salt as well as the Co^2+^ and Mn^2+^ complexes did not affect the composition of the reaction products. Through examination of selected parameters, it was revealed that the C-ON oxidation reaction for 4 h at 60 °C was satisfactory, producing AA with 71% selectivity and 100% C-ON conversion. A similar effect was observed when the reaction was conducted for 2 h at 60 °C and then for an additional 2 h at 80 °C. This approach was more advantageous for industrial processes due to dispersion of potentially hazardous energy. Shorter-chain acids, mainly GA and BA, were observed as the main by-products.

The developed catalytic system IPN/Co(acac)_2_/Mn(acac)_2_ successfully increased the selectivity of dicarboxylic acids determined from cyclic ketones. The type of oxidizing agent used (air or O_2_) did not affect the composition of the reaction products. However, on a larger scale, it was necessary to carry out the oxidation reaction in air due to safety reasons. The results indicate that the oxidation proceeds with a high energy effect, which requires a reactor with efficient cooling.

A block diagram was presented describing the preparation of dicarboxylic acids as a simple method requiring only a limited number of unit operations. The raw materials, solvents, and catalysts used were commercially available, and their prices were relatively low. The proposed method for the obtainment of AA from C-ON, using air as the oxidizing agent, was more environmentally friendly than other industrial methods, which employ HNO_3_ as an oxidant.

It is highly probable that in the next 5–10 years, the use of HNO_3_ as an oxidizing agent will no longer be necessary. The market report [71] for AA for 2020–2025 clearly emphasizes the expectations of stringent environmental regulations regarding the production process. These regulations are likely to inhibit market development and increase interest in technologies for the AA production, e.g., bio-based. It is estimated that new technologies will be implemented in the coming years. The chemical industry recognizes this threat and therefore conducts intensive research to improve the existing production methods or select new alternative production methods.

The proposed method for AA production is an interesting alternative to current industrial production methods based on HNO_3_. Certainly, the optimization of C-ON oxidation using the Co/Mn/IPN system can increase selectivity, which has economic benefits. Moreover, legal and environmental conditions related to the emission of greenhouse gases significantly influence the constantly growing price of AA on the world markets. Therefore, it is possible that current technologies for dicarboxylic acids formation may be considered unprofitable in the future and our method an interesting alternative to the traditional, non-ecological method.

## 5. Patents

The research results described in this article have become the subject of a Polish patent with the number PL 239347.

## Figures and Tables

**Table 1:** Review of used catalysis and conditions in the oxidation of cyclohexanone to AA with O_2_ or air.

Entry	Catalysis System	Solvent	Temp. (°C)	Pressure (MPa)	Time (h)	Conv. (%) ^a^	Sel. (%) ^b^	Ref.
1	Mn-HTS	-	90	0.6	9	68	93	[44]
2	Pt/carbon	H_2_O	140	5.0	-	100	39	[45]
3	Pt/carbon/monolith	H_2_O	140	5.0	1	100	21	[46]
4	Modified carbon material	H_2_O	140	5.0	6	100	33	[47]
5	Hybrid iron phosphonate material (FePO-1–2)	H_2_O	75	0.1	10	96	72	[48]
6	Mn-HTS	AcOH	90	0.6	9	91	86	[44]
7	NHPI/Mn(acac)_2_	AcOH	100	0.1	6	99	64	[26]
8	Co(OAc)_2_/NaBr	AcOH	80	0.1	1	-	36	[49]
9	Co(OAc)_2_	AcOH	105	0.5	-	98	71	[50]
10	POM Keggin	AcOH/H_2_O	70	1.0	6	18	65	[51]
11	Cluster Co/Mn/MEK ^[c]^	AcOH/H_2_O	100	3.8	8	98	87	[35]
12	H_5_[PMo_10_V_2_O_40_]·30H_2_O	AcOH/H_2_O	70	0.1	7	99	40	[52]
13	H_7_[PMo_8_V_4_O_40_]·12H_2_O	AcOH/H_2_O	70	0.1	7	99	51	[52]
14	Mn(OAc)_2_/Co(OAc)_2_/p-TS ^[d]^	AcOH/H_2_O	70	0.1	5	97	78	[53]
15	Mn(NO_3_)_2_/Co(NO_3_)_2_	AcOH/H_2_O	40	0.1	-	98	93	[54]
16	Nanosheets of Ni-SAPO-34 molecular Sieve	acetone	135	1.5	20	30	87	[55]

^a^, conversion C-ON; ^b^, AA selectivity; ^c^, methyl ethyl ketone; ^d^, para-toluenesulfonic acid.

**Table 2:** Aerobic oxidation of cyclohexanone in the presence of R-ONO/Co^2+^/Mn^2+^.

Entry	Initiator	Mn^2+^	Co^2+^	Conv. (%) ^a^	Sel. AA (%)	Sel. GA (%)
1	-	0.5	0.5	68	42	16
2	IPN	-	-	38	45	10
3	IPN	0.5	-	94	56	13
4	IPN	-	0.5	66	43	12
5	IPN	0.5	0.5	97	62	19
6	PN	0.5	0.5	96	43	14
7	TBN	0.5	0.5	89	51	16
8	HNO_3_	0.5	0.5	96	54	18
9 ^b^	IPN	0.5	0.5	95	59	19
10 ^c^	IPN	0.5	0.5	92	58	18

C-ON 2 mL (19.3 mmol), AcOH 20 mL (349.7 mmol), initiator 10 mol%, Co(acac)_2_ 0.5 mol%, Mn(acac)_2_ 0.5 mol%, 60 °C, O_2_ 0.5 MPa, 2 h; ^a^, conversion of C-ON; ^b^, Co(NO_3_)_2_ and Mn(NO_3_)_2_; ^c^, Co(OAc)_2_ and Mn(OAc)_2_.

**Table 3:** Influence of selected parameters on the composition of C-ON oxidation reaction products.

Entry	Solvent	Temp. (°C)	Pressure (MPa)	Time (h)	Conv. (%) ^a^	Sel. AA (%)	Sel. GA (%)
1	AcOH	40	0.5	2	52	31	10
2	AcOH	50	0.5	2	91	41	11
3	AcOH	60	0.5	2	97	62	19
4	AcOH	80	0.5	2	100	68	18
5	AcOH	100	0.5	2	99	59	21
6 ^b^	AcOH	60–80	0.5	2	99	67	23
7	AcOH	60	0.1	2	83	61	17
8	AcOH	60	1.0	2	99	63	21
9	AcOH	60	1.5	2	97	64	21
10 ^c^	-	100	0.5	2	35	30	8
11	MeCN	100	0.5	2	99	31	16
12	PhCN	100	0.5	2	99	64	20
13	AcOH	40	0.5	2	52	31	10

C-ON 2 mL (19.3 mmol), solvent 20 mL, IPN 10 mol%, Co(acac)_2_ 0.5 mol%, Mn(acac)_2_ 0.5 mol%, and O_2_ at 2 h; ^a^, conversion of C-ON; ^b^, 1 h at 60 °C and 1 h at 80 °C; ^c^, C-ON 20 mL (193 mmol).

**Table 4:** Oxidation of cyclohexanone, cyclohexanol, and cyclohexane.

Entry	Raw Material	Time (h)	Conv. (%) ^a^	Sel. AA (%)	Sel. C-ON (%)	Sel. GA (%)
1	C-ON	2	97	62	-	19
2	C-ON	6	100	70	-	23
3	C-OL	2	95	11	47	5
4	C-OL	6	98	20	51	6
5 ^b^	C-OL or C-ON	2	100	58	-	17
6 ^b^	C-OL or C-ON	6	100	60	-	18

Substrate 2 mL, AcOH 20 mL (349.7 mmol), Co(acac)_2_ 0.5 mol%, Mn(acac)_2_ 0.5 mol%, IPN 10 mol%, 60 °C, and O_2_ 0.5 MPa; ^a^, conversion; ^b^, retro m:m 1:1.

**Table 5:** Oxidation of cyclic ketones to dicarboxylic acids.

Entry	Temp. (°C)	IPN (% mol)	Raw Material	Conv. (%) ^a^	Sel. Main Product (%)	Sel. By-Product (%)
			Cyclopentanone		Glutaric acid	Succinic acid
1	80	-		100	33	9
2	80	10		100	47	11
3	60	10		93	20	3
			Cyclohexanone		Adipic acid	Glutaric acid
4	80	-		100	62	17
5	80	10		100	70	18
6	60	10		100	71	23
			Cycloheptanone		Pimelic acid	Adipic acid
7	80	-		87	52	15
8	80	10		99	54	18
9	60	10		47	15	3
			Cyclooktanone		Suberic acid	Pimelic acid
10	80	-		98	62	14
11	80	10		100	70	12
12	60	10		97	54	18
			Cyclododecanone		Dodecane-1,12-dioic	Undecane-1,11-dioic acid
13	80	-		75	44	10
14	80	10		100	59	9
15	60	10		100	53	3
			2-Methyl cyclohexanone		6-Oxyheptanoic acid	Glutaric acid
16	80	-		50	44	16
17	80	10		100	68	14
18	60	10		100	69	14
			3-Methyl cyclohexanone		3-Methyladipic acid	2-Methyladipic acid
19	80	-		69	45	18
20	80	10		100	64	16
21	60	10		100	59	16
			4-Methyl cyclohexanone		3-Methyladipic acid	2-Methylglutaric acid
22	80	-		68	61	9
23	80	10		100	88	10
24	60	10		99	75	8

Substrate 2 mL, AcOH 20 mL (349.7 mmol), Co(acac)2 0.5 mol%, Mn(acac)2 0.5 mol%, initiator 10 mol% at 60 °C, and O_2_ 0.5 MPa at 2 h; ^a^, conversion.

## Data Availability

All data are available in the manuscript or upon request to the corresponding author.
